# The Value of Tracheal Visualization in Tracheostomized Patients in Skilled and Long-Term Care Homes

**DOI:** 10.7759/cureus.76748

**Published:** 2025-01-01

**Authors:** Gustavo Ferrer, César Alas-Pineda, Viviane Manara, Mari Tesch, Kristhel Gaitán-Zambrano, Dennis J Pavón-Varela

**Affiliations:** 1 Department of Pulmonary and Critical Care Medicine, Aventura Hospital and Medical Center, Aventura, USA; 2 Department of Analytics, Ferrer Pulmonary Institute, Hallandale Beach, USA; 3 Department of Pulmonary and Critical Care Medicine, Ferrer Pulmonary Institute, Hallandale Beach, USA; 4 Department of Research and Development, Dr. Ferrer BioPharma, Hallandale Beach, USA

**Keywords:** airway management, long-term care, retrospective study, skilled nursing facilities, tracheal visualization, tracheostomy, weaning

## Abstract

Objective

This study aims to assess the impact of tracheal visualization on weaning success among tracheostomized patients in skilled and long-term care facilities, highlighting its role in reducing complications and enhancing clinical outcomes.

Methods

A retrospective observational study was conducted on tracheostomized patients residing in skilled nursing homes in Florida between 2018 and 2023. The study included individuals aged 18 years or older with established tracheostomies. Routine tracheal visualization techniques were used to confirm tube placement, evaluate tracheal health, and detect complications such as infections, inflammation, or obstructions.

Results

Among the 21 patients analyzed, a weaning success rate of 23.8% was observed. Patients who successfully weaned had fewer comorbidities and higher tracheal health scores compared to those who did not. Routine tracheal visualization reduced complications related to tube malposition and facilitated timely interventions, such as correcting tube displacement or managing airway obstructions.

Conclusions

This study underscores the significance of incorporating tracheal visualization into care protocols for tracheostomized patients, especially in skilled nursing and long-term care settings. Future research should focus on developing standardized protocols to improve care quality and ensure patient safety, particularly in resource-constrained environments.

## Introduction

Tracheostomy is a common surgical procedure performed on patients requiring prolonged mechanical ventilation or experiencing upper airway obstruction. It involves creating an opening in the trachea to insert a tracheostomy tube, ensuring adequate ventilation. Proper management and regular assessment of the tracheostomy tube are essential to patient safety, as poorly maintained tracheostomies can lead to complications such as infections, tube displacement, or obstructions [1,2].

Tracheal visualization has become a cornerstone in the clinical management of tracheostomized patients, enabling clinicians to directly inspect the airway, ensure proper tube positioning, and detect potential complications early [3,4]. Techniques like flexible laryngoscopy are particularly beneficial for tracheostomized patients, who often have comorbid conditions that compromise respiratory function, creating complex clinical scenarios [5].

Continuous monitoring of the tracheostomy site and the patient’s respiratory parameters, such as oxygen saturation and lung sounds, is vital for identifying issues before they escalate [6,7]. Studies indicate that regular airway visualization, combined with clinical assessments, significantly reduces complication rates [8,9]. This approach is especially critical during the weaning process, where patients transition from mechanical ventilation to spontaneous breathing.

The weaning process poses unique challenges, requiring careful evaluation of a patient’s ability to breathe independently. Tracheal visualization facilitates this transition by providing real-time insights into the airway’s condition and identifying barriers to successful weaning [10]. Early detection of complications during weaning can improve the likelihood of success and minimize the risk of reintubation [11].

In long-term care settings, such as nursing homes (NHs), healthcare providers often contend with limited resources and less frequent specialist visits. In these environments, regular tracheal visualization becomes even more crucial for maintaining the health and safety of tracheostomized patients [12,13]. Flexible laryngoscopy and other visualization techniques equip healthcare providers with valuable tools to manage these patients effectively, improving clinical outcomes [14,15].

The emotional and psychological impacts of being tracheostomized should not be overlooked. Many patients experience stress and anxiety related to their dependence on mechanical ventilation and the risk of complications [16]. Comprehensive care that includes regular airway monitoring can help alleviate these concerns, improving the overall quality of life for tracheostomized individuals [17,18].

Tracheostomy is a common intervention for patients requiring prolonged mechanical ventilation or those with upper airway obstruction. Proper maintenance and assessment of tracheostomies are essential for preventing complications and ensuring patient safety [19,20]. While tracheal visualization plays a crucial role in acute care, its application in long-term care settings remains underexplored. Residents in skilled NHs face unique challenges, such as limited resources and complex comorbidities, which can impact clinical outcomes [21-24].

This study aims to address this knowledge gap by evaluating the effectiveness of tracheal visualization in improving weaning success among tracheostomized patients in long-term care facilities. In doing so, it seeks to contribute to the development of enhanced care practices that can improve patient outcomes and quality of life. The study specifically focuses on patients residing in skilled and long-term care NHs, where such interventions are particularly relevant due to the challenges associated with providing continuous, specialized care.

## Materials and methods

This is a quantitative, cross-sectional, observational, analytical, and retrospective study aimed at evaluating the effectiveness of tracheal visualization in the weaning process of tracheostomized patients in long-term care homes in Florida, USA. Outcomes were assessed retrospectively by analyzing existing medical records of tracheostomized patients treated in long-term care homes between 2018 and 2023, focusing on the visualization techniques employed and the subsequent success or failure of the weaning process, without active manipulation of patient care. The study employed a non-probabilistic convenience sampling method, selecting patients from those available in the NHs during the 2018-2023 period.

The study included patients aged 18 years or older who had a tracheostomy and were residing in a long-term care home in Florida, USA, between 2018 and 2023. Additionally, patients with no acute severe pulmonary illness or underlying conditions requiring hospitalization, and who had a stable health status that allowed for the evaluation of their tracheostomy and participation in the study, were included.

Patients were excluded if they had terminal illnesses or conditions that contraindicate weaning, such as advanced cancer or chronic respiratory diseases in their terminal stages. Other exclusion criteria included medical contraindications for using tracheal visualization techniques, such as anatomical abnormalities that prevent the safe use of flexible laryngoscopy, recent major surgeries (within the past 30 days) that could interfere with respiratory stability or participation in tracheal visualization, and active respiratory infections or septicemia that could complicate their clinical stability during the weaning process.

The study included various key variables to assess the clinical profile of the patients and the weaning process. Demographic variables such as age, sex, race, religion, and marital status, along with admission information, were collected. The dependent variables of the study included successful weaning, defined as the patient's ability to remain disconnected from mechanical ventilation for ≥48 hours while maintaining a patent airway without the need for reintubation. The Murray Score, which assesses airway inflammation, mobility of cartilage, and ulceration, was also used to quantify tracheobronchial health. The score was graded to reflect the condition of the airways.

The independent variables considered included various clinical characteristics, such as tracheostomy tube characteristics (type, size, and features) and laryngoscopy management interventions (inhalers, head elevation, steroid courses, nasal spray, nebulization, or oral hygiene). The weaning process was categorized as either successful or unsuccessful, with specific reasons documented for unsuccessful cases. Successful weaning was defined as achieving ≥48 hours of independent breathing with stable oxygenation following the removal of the tracheostomy tube. Failed weaning was defined as the need for reintubation or failure to meet the criteria for independent breathing.

Laryngoscopy allowed for a detailed assessment of inflammation, tracheal mobility, cartilage integrity, and the presence of ulcerations, which were reflected in the Murray Score, used to evaluate airway condition with a maximum score of 8 points. However, as the Murray Score has not been validated for post-acute care settings, Dr. Ferrer and his team, based on their extensive experience managing long-term tracheostomy patients, developed a new scoring system that focuses on the long-term consequences of tracheostomy. This new system added granulomas, nodules, and masses, resulting in a total score of 10 points. The study also evaluated the presence of comorbidities, including dysphagia post-percutaneous endoscopic gastrostomy (PEG), chronic respiratory failure post-tracheostomy, hypertension, type 2 diabetes mellitus (T2DM), coronary artery disease (CAD), and chronic obstructive pulmonary disease (COPD). Potential confounding variables identified included advanced age, the total number of comorbidities, a history of previous respiratory diseases, and the patient’s overall functional status. Statistical adjustments were made to control for these factors in the analysis.

During the tracheal visualization process, a flexible video laryngoscope was utilized, enabling healthcare professionals to directly assess the patency of the tracheal cannula as well as the condition of both the upper and lower airways. This procedure was performed under light sedation to ensure patient comfort, allowing for thorough inspections to detect signs of inflammation, ulceration, granuloma formation, and other potential complications that could interfere with the weaning process. Additionally, laryngoscopy facilitated the verification of vocal cord movement and the assessment of the anatomical integrity of the trachea - both critical factors for ensuring successful weaning.

The data collection process was standardized to minimize information bias, and all patients who met the inclusion criteria during the study period were included to avoid selection bias. Data were analyzed using IBM SPSS Statistics for Windows, Version 27.0 (Released 2020; IBM Corp., Armonk, NY, USA). Descriptive analyses of the sample characteristics were performed, followed by inferential tests to compare variables between groups of patients who successfully weaned and those who did not. The Mann-Whitney U test was used for comparisons between groups, as the distribution of some variables did not follow a normal distribution. The statistical significance level was set at p ≤ 0.05. Specific comparisons were made between the subgroups of patients who successfully weaned and those who did not, focusing on the prevalence of comorbidities and the characteristics of the tracheostomy tube.

The study protocol was reviewed and approved by the Ethics Committee of Beyond Bound under approval number #2024-01, ensuring compliance with ethical principles outlined in the Declaration of Helsinki. As a retrospective study based on pre-existing medical records, the requirement for informed consent was waived by the Ethics Committee. All data were anonymized to protect patient confidentiality, and no identifiable information was included in the analysis. Institutional permissions were obtained from each participating facility to access and analyze patient records in accordance with ethical and regulatory guidelines.

This study presents several limitations that should be considered when interpreting its findings. As a retrospective observational study, it relies on medical records, which may be incomplete or inconsistent, introducing potential information bias. The small sample size and focus on long-term care facilities in a specific geographic region limit the generalizability of the results. Selection bias may have occurred due to the exclusion of patients with incomplete records, and residual confounding factors, such as variations in care quality, could influence outcomes.

The lack of standardized protocols for tracheal visualization techniques introduces variability, and the retrospective design limits the ability to establish causal relationships. Observer bias during data extraction and the absence of psychosocial factors in the analysis further restrict the study's scope. Additionally, the adaptation of the Murray Score, originally validated for acute lung injury, may require further validation in this context. Future research should address these limitations through prospective designs, larger sample sizes, and standardized interventions.

## Results

Patient characteristics

**Figure 1:**
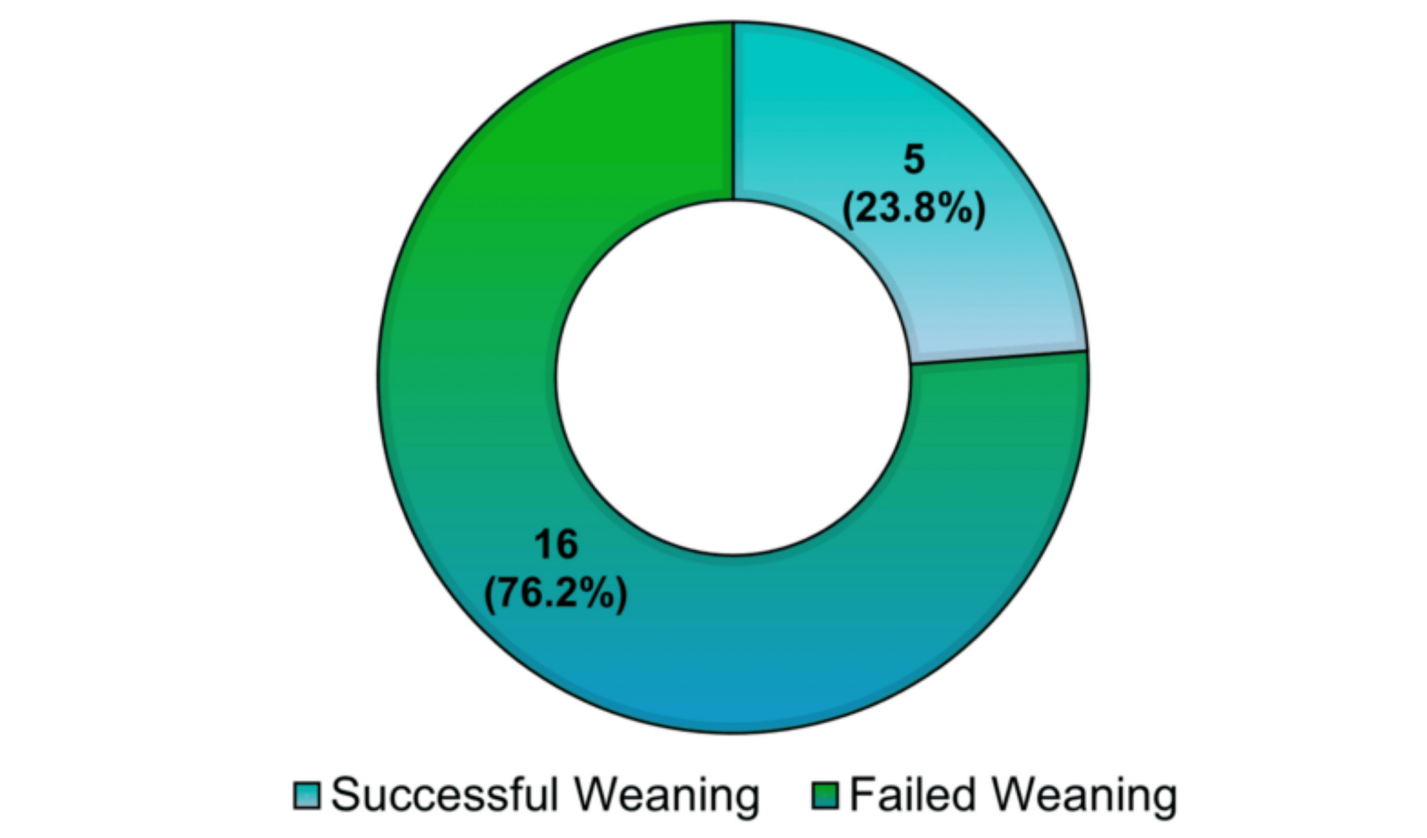
Distribution of weaning outcomes among tracheostomy patients

A total of 21 patients with tracheostomy were analyzed at an NH to evaluate the role of tracheal visualization in the weaning process. The median age of the patients was 74 years (IQR: 66-86.5), with ages ranging from 42 to 92 years. The majority of patients were male (66.7%), and the predominant race was Caucasian (76.2%). Further sociodemographic data are presented in Table 1. Of the patients, five (23.8%) successfully underwent weaning. The management of the visualization technique was adequate, and patients who successfully weaned demonstrated clinical improvement by tolerating ≥48 hours of spontaneous breathing without additional ventilatory support while maintaining a patent airway. The remaining 76.2% failed the weaning process (Figure 1), either due to complications during weaning or failure to meet the established criteria. No re-intubations were reported in the successfully weaned group after the ≥48-hour period.

**Table 1:** Patient demographics and comorbidities ^a ^Median; SD ^b^ Other conditions: acute renal failure, atrioventricular block, atrial fibrillation, Alzheimer’s disease, congestive heart failure, cerebrovascular accident, dementia, hypothyroidism, intracerebral hemorrhage, obesity, Parkinson’s disease, sick sinus syndrome, seizure disorder, depression, encephalopathy, aortic valve dysfunction, paraplegia, and tracheomalacia CAD, coronary artery disease; COPD, chronic obstructive pulmonary disease; ESRD, end-stage renal disease

Variable	Total patients (n = 21)	Patients with trach (n = 21)		p-value
		Patients with successful weaning (n = 5)	Patients with failed weaning (n = 16)	
Patient age, median^a^	74 (66.0-86.5)	84 (69.0-86.5)	72 (66.0-88.0)	
<65 years	3 (14.3)	0 (0.0)	3 (14.3)	0.296
≥65 years	18 (85.7)	5 (23.8)	13 (61.9)	
Sex				
Female	10 (47.6)	2 (9.5)	8 (38.1)	0.550
Male	11 (52.4)	3 (14.3)	8 (38.1)	
Race				
African American	3 (14.3)	0 (0.0)	3 (14.3)	0.659
Hispanic	6 (28.6)	2 (9.5)	4 (19.0)	
White	12 (57.1)	3 (14.3)	9 (42.9)	
Religion				
Baptist	2 (9.5)	1 (4.8)	1 (4.8)	0.340
Catholic	6 (28.9)	2 (9.5)	4 (19.0)	
Christian	1 (4.8)	1 (4.8)	0 (0.0)	
Jehovah’s Witness	1 (4.8)	0 (0.0)	1 (4.8)	
None	3 (14.3)	0 (0.0)	3 (14.3)	
No data	8 (38.1)	1 (4.8)	7 (33.3)	
Marital status				
Married	6 (28.6)	3 (14.3)	3 (14.3)	0.122
Single	4 (19.0)	0 (0.0)	4 (19.0)	
No data	11 (52.4)	2 (9.5)	9 (42.9)	
Comorbidity (>6)				
Yes	12 (57.1)	4 (19.0)	8 (38.1)	0.237
No	9 (42.9)	1 (4.8)	8 (38.1)	
History of respiratory failure				
Yes	2 (9.5)	0 (0.0)	2 (9.5)	0.406
No	19 (90.5)	23.8 (5)	14 (66.7)	
Respiratory failure after trach				
Yes	18 (85.7)	5 (23.8)	13 (61.9)	0.296
No	3 (14.3)	0 (0.0)	3 (14.3)	
Predominant clinical condition^b^				
Dysphagia	19 (90.5)	5 (23.8)	14 (66.7)	0.406
Chronic respiratory failure	18 (85.7)	5 (23.8)	13 (61.9)	0.296
Hypertension	16 (76.2)	4 (19.0)	12 (51.1)	0.819
Diabetes mellitus	11 (52.4)	3 (14.3)	8 (38.1)	0.696
CAD	4 (19.0)	1 (4.8)	3 (14.8)	0.950
COPD	4 (19.0)	1 (4.8)	3 (14.8)	0.950
ESRD	3 (14.3)	1 (4.8)	2 (9.5)	0.676

The admission diagnosis for all patients was respiratory failure. Each patient had at least one comorbidity or concomitant condition, with the majority presenting multiple medical conditions. Specifically, 38.1% of patients had six comorbidities, 23.8% had five, and 14.3% had seven. The most prevalent comorbidities in the cohort included dysphagia post-PEG (90.5%), chronic respiratory failure post-tracheostomy (85.7%), hypertension (76.2%), T2DM (52.4%), CAD (19.0%), and COPD (19.0%). These conditions were consistently observed across patients, regardless of the total number of comorbidities (Table 1).

Laryngoscopy management strategies

Our study assessed various laryngoscopy management strategies for patients with tracheostomy in an NH setting. These strategies included the use of nebulizers, head elevation, oral hygiene, nasal sprays, and inhalers. A total of 61.9% of patients (13 patients) required some form of intervention, with head elevation being the most common (32.4%), followed by nebulization (29.4%), oral hygiene (29.4%), and the use of inhalers, steroid courses, and nasal sprays, each accounting for 2.9%. These interventions play a critical role in weaning management as they can impact airway inflammation and mobility (Table 2).

**Table 2:** Laryngoscopy management and intervention outcomes by weaning success

Management	Patients with trach (n = 21)			p-value
	Total patients (n = 21)	Patients with successful weaning (n = 5)	Patients with failed weaning (n = 16)	
Laryngoscopy management				
Inhalers	1 (4.8)	0 (0.0)	1 (4.8)	0.762
Head elevation	11 (52.4)	2 (9.5)	9 (42.9)	0.450
Steroid course	1 (4.8)	1 (4.8)	0 (0.0)	0.238
Nasal spray	1 (4.8)	0 (0.0)	1 (4.8)	0.762
Nebulization	10 (47.6)	2 (9.5)	8 (38.1)	0.550
Oral hygiene	10 (47.6)	2 (9.5)	8 (38.1)	0.550

Tracheostomy tube characteristics

The characteristics of tracheostomy tubes were evaluated at both the time of admission (trach on admission) and during the hospital stay (trach inpatient) in an NH setting. At admission, 66.7% of patients had a double-cuffed tube (DCT), 4.8% had an extra-long tube (XLT), and 9.5% used a Shiley tube, with data unavailable for 19.0% of patients. Regarding tube size, 66.7% of patients had size six tubes, 14.3% had size eight tubes, and 19.0% had missing data. In terms of tube features, 47.6% of the tubes were non-fenestrated, 28.6% were fenestrated, and 4.8% were both T/C and fenestrated, with data unavailable for 19.0% of cases.

During the hospital stay, the distribution of tracheostomy tube types shifted. A total of 66.7% of patients used Shiley tubes, while only 9.5% maintained DCTs and 4.8% continued with XLTs, with data unavailable for 19.0% of patients. In terms of tube size, 76.2% of patients used size 6 tubes, and 4.8% used size 8 tubes, with 19.0% missing data. Tube features also varied: 42.9% of the tubes were fenestrated, 28.6% were non-fenestrated, and 4.8% were both T/C and fenestrated, with 23.8% of cases lacking data.

Patterns of weaning success

Among patients with successful weaning, the mean age was 79.0 ± 9.2 years, whereas those with failed weaning had a mean age of 72.8 ± 14.6 years. No significant differences in sex or race were observed between the groups with successful and failed weaning (Table 1). The most common reasons for failed weaning were issues during the weaning process (such as secretions or bleeding) (23.8%) or failure to meet the weaning criteria (52.4%) (p > 0.05; statistical test: Mann-Whitney U test).

In terms of comorbidities, 38% of patients with failed weaning had more than six comorbidities compared to 19.0% in the successful weaning group. Chronic respiratory failure was prevalent in 61.9% of patients with failed weaning and 23.8% of those with successful weaning, while dysphagia was present in 66.7% of failed weaning patients and 23.8% of successful weaning patients. These were the most prevalent clinical conditions.

The weaning process was categorized for all patients, with 23.8% achieving successful weaning. Patients who successfully weaned showed better scores on the Murray Score, an index that evaluates inflammation, mobility, ulceration, and cartilage of the airway (25). The individual components of the Murray Score showed a mean of 1.05 for inflammation, 1.48 for mobility, and 0.95 for cartilage. No ulcerations were observed in any patients. It was noted that those with failed weaning had a slightly higher median Murray Score of 4.0 (IQR: 3.0-4.0) compared to successful weaning patients, who had a median score of 3.0 (IQR: 3.0-5.0), indicating a higher burden of comorbidities. Among patients with failed weaning, 23.8% had a score of 3 points, while 14.6% of patients with successful weaning also had a score of 3 points (Table 3). These scores suggest that a higher burden of comorbidities and complications is associated with a lower likelihood of successful weaning in patients with tracheostomy (Table 4).

**Table 3:** Characteristics of patients with successful weaning CAD, coronary artery disease; COPD, chronic obstructive pulmonary disease; DCT, double-cuffed tube; DM, diabetes mellitus; ESRD, end-stage renal disease; HTN, hypertension

Patient no.	Sex	Age	Clinical conditions	Trach tube (admission/inpatient)	No. of tubes	Tube feature	Total score	Total comorbidities
1	M	87	COPD, DM, dysphagia, HTN, Parkinson’s disease, and chronic respiratory failure	DCT/No data	8	Fenestrated	3	6
2	M	68	Acute renal failure, cerebrovascular accident, chronic respiratory failure, DM, dysphagia, and HTN	DCT/Shiley	6	Not fenestrated	3	6
3	M	70	CAD, chronic respiratory failure, DM, dysphagia, ESRD, and HTN	DCT/Shiley	6	Fenestrated	3	6
4	F	84	Chronic respiratory failure, dysphagia, and tracheomalacia	DCT/DCT	6	Fenestrated	5	3
5	F	86	Alzheimer’s disease, chronic respiratory failure, dysphagia, HTN, hypothyroidism, sick sinus syndrome, and aortic valve dysfunction	DCT/Shiley	6	Not fenestrated	5	7

**Table 4:** Total score (inflammation, mobility, cartilage, ulceration + granuloma, nodule, and mass score) ^a^ Murray Score, including additional points for granulomas, nodules, and masses (2 points).

Total score (10 points)^a^	Patients with trach (n = 21)		Total patients (n = 21)
	Patients with successful weaning (n = 5)	Patients with failed weaning (n = 16)	
Total score, mean^a^	3.0 (3.0-5.0)	4.0 (3.0-4.0)	4 (3.0-4.5)
2 points	0 (0.0)	2 (9.5)	2 (9.5)
3 points	3 (14.6)	5 (23.8)	8 (38.1)
4 points	0 (0.0)	6 (28.6)	6 (28.6)
5 points	2 (9.5)	3 (14.3)	5 (23.8)

Figure 1 illustrates the distribution of weaning outcomes among tracheostomy patients treated at the NH. Of the total patients, 23.8% (five patients) achieved successful weaning, while 76.2% (16 patients) did not meet the criteria for weaning.

**Figure 2:**
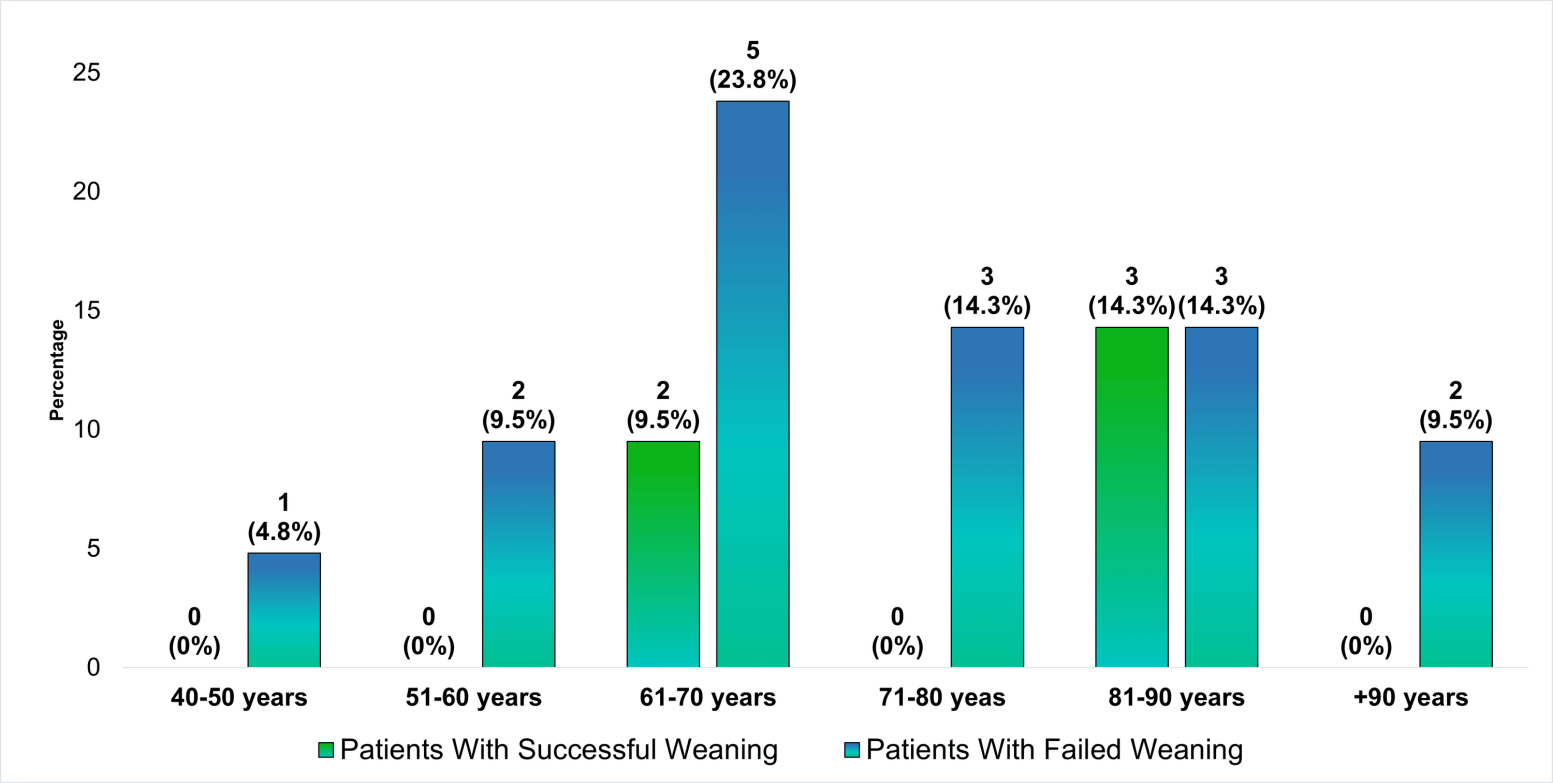
Age distribution and weaning success in tracheostomy patients

Figure 2 depicts the age distribution of tracheostomy patients in relation to their weaning outcomes. It highlights the percentage of patients who successfully weaned versus those who failed, across age groups ranging from 40 to over 90 years. Successful weaning was most prevalent in the 61-70 years age group, while higher rates of failed weaning were observed in the older age groups.

**Figure 3:**
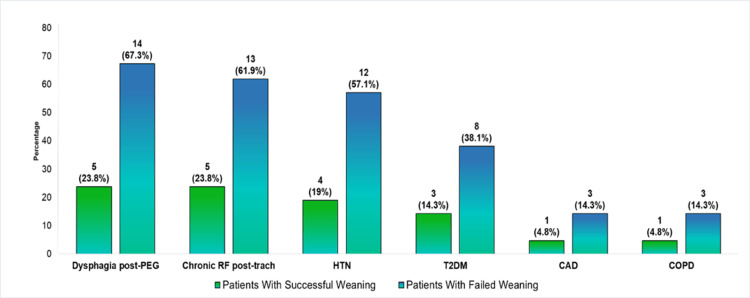
Distribution of predominant clinical conditions in patients with tracheostomy by weaning success CAD, coronary artery disease; chronic RF post-trach, chronic respiratory failure after tracheostomy; COPD, chronic obstructive pulmonary disease; dysphagia post-PEG, dysphagia after percutaneous endoscopic gastrostomy; HTN, hypertension; T2DM, type 2 diabetes mellitus

Figure 3 illustrates the distribution of the most common clinical conditions among tracheostomy patients, comparing those who achieved successful weaning with those who failed. The chart shows that dysphagia post-PEG is the most prevalent condition, occurring in 67.3% of patients who failed to wean, compared to 23.8% of patients who were successfully weaned. Similarly, chronic respiratory failure post-tracheostomy was present in 61.9% of patients with failed weaning, whereas only 23.8% of successfully weaned patients had this condition.

Hypertension (HTN) was present in 57.1% of patients who failed weaning, compared to 19% in the successful weaning group. Similarly, T2DM was more prevalent in the failed weaning group (38.1%) than in the successful weaning group (14.3%).

## Discussion

This study underscores the importance of tracheal visualization in the management and weaning of tracheostomized patients in skilled nursing and long-term care settings. With a weaning success rate of 23.8%, our findings highlight the value of tracheal visualization in enhancing care quality, enabling early complication detection, and improving outcomes in resource-limited environments. This research is novel in applying established tracheal care principles, typically explored in acute care settings, to long-term care facilities.

Proper tracheal tube placement is crucial for effective ventilation, preventing complications, and ensuring patient safety. Correct tube placement supports adequate oxygenation and ventilation, especially for patients relying on mechanical ventilation. Incorrect placement, such as esophageal intubation, can result in inadequate ventilation, hypoxia, and life-threatening complications like brain damage or cardiac arrest [1,3,25-27]. Additionally, correct placement reduces the risk of aspiration, which can lead to pneumonia and severe respiratory distress, as emphasized in previous tracheostomy care studies [4,6,26].

In surgical and emergency care settings, precise tube placement ensures airway patency and effective respiratory management. Freeman highlights that accurate tube placement minimizes complications such as pneumothorax and tracheal stenosis [25]. While advanced placement methods like ultrasound guidance may be unavailable in resource-constrained long-term care settings, our findings suggest that routine tracheal visualization can serve as a practical alternative to ensure proper tube positioning and improve patient outcomes [25].

Tracheal visualization is vital in tracheostomy management, offering real-time insights into airway patency, tube condition, and early signs of complications such as tube displacement or obstruction. Our study showed that routine tracheal visualization allowed for the timely confirmation of tube placement and facilitated early intervention for issues like inflammation or infection, in line with Gobatto et al.’s emphasis on precision in tracheostomy procedures [6].

Beyond confirming correct tube placement, visualization techniques offer valuable information on respiratory status and oxygenation. For example, abnormal tracheal movement observed during visualization may indicate airway obstruction, prompting immediate corrective action. Monitoring and responding to such complications improves patient safety and reduces weaning failure rates [27].

Our results are consistent with the WEAN SAFE study, which identified delays in initiating weaning and excessive sedation as significant predictors of weaning failure [25]. Routine tracheal visualization, as employed in our study, addresses these challenges by enabling prompt interventions, minimizing delays, and supporting the weaning process. Kumar et al. also noted that complications like granuloma formation, infection, and tube obstruction negatively impact weaning success [27]. Our findings align with this, as routine visualization allowed for the early detection and management of such complications, ultimately improving patient outcomes.

Furthermore, integrating tracheal visualization into standard practice in skilled NHs addresses the challenges posed by variable resources, providing a cost-effective solution to enhance care quality [25,27]. Tài et al. emphasized the importance of standardized respiratory assessments in improving weaning outcomes [28]. By incorporating tracheal visualization into routine care, our study extends these principles to long-term care facilities, where practice variability often complicates patient management. This novel application demonstrates the adaptability of tracheal visualization techniques to diverse healthcare environments.

The inclusion of tracheal visualization in skilled nursing facilities offers a cost-effective solution to challenges in tracheostomy management. While advanced imaging technologies may not be feasible in these settings, routine visualization provides a practical means of enhancing care quality. By facilitating early detection and management of complications, these practices improve patient outcomes and reduce healthcare burdens.

Implementing comprehensive management programs, like the one proposed in this study, not only enhances clinical outcomes but also yields significant economic benefits. Published data suggest that the cost of supplemental oxygen in long-term care settings can be substantial if its use is unnecessarily prolonged [29]. For tracheostomized patients, who typically remain in NHs for approximately 90 days, a proactive surveillance approach during the first week of admission could identify patients who do not require continued supplemental oxygen [30]. This approach could reduce oxygen usage in the remaining 70 days, resulting in considerable cost savings. Such strategies alleviate financial burdens and optimize healthcare resource allocation, promoting a more efficient and sustainable care model [29,30].

While this study emphasizes the value of tracheal visualization, several limitations must be considered. The retrospective design restricts our ability to establish causality, and the small sample size may limit the generalizability of our findings. Future research should use prospective designs with larger cohorts to validate these results. Additionally, exploring the implementation of standardized visualization protocols across long-term care facilities could provide further insights into improving tracheostomy care.

We propose a systematic validation process for the newly developed Ferrer Long-Term Tracheostomy Assessment (Ferrer Score) to confirm its reliability and clinical utility in evaluating long-term outcomes for tracheostomized patients. The validation will involve prospective multicenter studies with larger and more diverse patient populations to ensure generalizability. The Ferrer Score, designed to address gaps not covered by existing tools like the Murray Score, incorporates parameters specific to long-term tracheostomy care, such as granulomas, nodules, and mass formations. The validation process will include inter-rater reliability assessments, sensitivity analyses, and comparisons with existing metrics to evaluate its predictive value for weaning success, complication rates, and overall patient outcomes. By rigorously testing the score in multiple healthcare settings, we aim to establish it as a standardized tool to improve decision-making and care protocols for tracheostomized patients.

Limitations

This study has several limitations that should be acknowledged for proper interpretation of the findings. As a retrospective observational study, it relies on existing medical records, which may be incomplete or inconsistent. The sample size is small, and the study is limited to tracheostomized patients residing in skilled and long-term care facilities within a specific geographic region. The use of convenience sampling introduces selection bias, as only patients with well-documented medical histories were included. Finally, the newly developed Ferrer Score, designed to assess long-term tracheostomy outcomes, requires prospective validation to confirm its utility, reliability, and correlation with clinical outcomes across diverse patient populations.

## Conclusions

This study highlights the critical role of tracheal visualization in improving care outcomes for tracheostomized patients in skilled and long-term care facilities. Our findings underscore the importance of incorporating tracheal visualization into routine clinical practices to facilitate successful weaning and reduce complications such as airway obstruction, infection, and aspiration. By adopting these practices, long-term care facilities can better address the unique challenges faced by tracheostomized patients, ultimately improving their outcomes and quality of life. Future research should build on these findings by investigating the implementation of standardized protocols and assessing their impact across diverse healthcare settings.
