# Correction: The Value of Tracheal Visualization in Tracheostomized Patients in Skilled and Long-Term Care Homes

**DOI:** 10.7759/cureus.c456

**Published:** 2026-06-29

**Authors:** Gustavo Ferrer, César Alas-Pineda, Viviane Manara, Mari Tesch, Kristhel Gaitán-Zambrano, Dennis J Pavón-Varela

**Affiliations:** 1 Department of Pulmonary and Critical Care Medicine, Aventura Hospital and Medical Center, Aventura, USA; 2 Department of Analytics, Ferrer Pulmonary Institute, Hallandale Beach, USA; 3 Department of Pulmonary and Critical Care Medicine, Ferrer Pulmonary Institute, Hallandale Beach, USA; 4 Department of Research and Development, Dr. Ferrer BioPharma, Hallandale Beach, USA

This article has been corrected to fix the following typo: in the final paragraph of the Discussion section (prior to the Limitations subsection), the word “glaucoma” has been corrected to “granulomas”.

